# Coping mechanisms of bullying survivors: Experiences of secondary school learners at Mamusa sub-district in the North West province, South Africa

**DOI:** 10.4102/hsag.v31i0.3247

**Published:** 2026-07-09

**Authors:** Kgalaletso V. Mokeng, Salaminah Moloko-Phiri, Lwazi Tilolo

**Affiliations:** 1 Department of Quality in Nursing and Midwifery (NuMIQ), Faculty of Health Sciences, North-West University, Mafikeng, South Africa

**Keywords:** bullying, coping mechanisms, experiences, learners, victimisation

## Abstract

**Background:**

Bullying in schools has become a public health concern as it continues escalating and causing mental health issues, such as depression. Thus, victims of bullying apply various coping mechanisms for self-defence against bullies.

**Aim:**

This study explores and describes coping mechanisms used by bullied survivors of the specific school.

**Setting:**

Interviews were conducted at the participants’ homes in the Mamusa sub-district in Dr Ruth Segomotsi Mompati district in the North West province, South Africa.

**Methods:**

A qualitative research approach following exploratory, descriptive and contextual designs was used. Twelve participants were selected using purposive sampling. Data were collected through in-depth individual face-to-face unstructured interviews until data saturation. Thematic analysis, following Braun and Clarke’s six-step process, was used to analyse the data. Principles of ethical consideration were adhered to, and trustworthiness was ensured through credibility, dependability, confirmability, transferability and authenticity.

**Results:**

Two themes emerged: positive and negative coping mechanisms with six sub-themes.

**Conclusion:**

The findings revealed that victims used positive and negative coping mechanisms to deal with school bullying, which negatively affected the well-being of victims both academically and socially. Furthermore, extensive awareness and education on bullying needs to be promoted in schools to empower victims and learners. Concurrently, education on school bullying needs to be incorporated into nursing education for the proper management of victims.

**Contribution:**

The new information resulting from this research may improve teaching and nursing education regarding how nurses and teachers should be trained on the prevention of bullying at schools. Extensive awareness and education on anti-bullying policies need to be emphasised in schools to deal with school bullying.

## Introduction

The prevalence of school bullying has increased worldwide. The United Nations Educational, Scientific and Cultural Organisation (UNESCO 2019) reports that 32% of learners worldwide have been victims of bullying. In Jordan (Asia), bullying victimisation is at 7% and being a bully at 7.6% (Dardas et al. 2022). Hong Kong’s prevalence of bullying is at 38.6%, where participants reported having bullied others once or twice in the first wave of testing well-being of victims on bullying perpetration, and 42.4% of the participants reported being involved in bullying behaviour once or twice in the second wave of the study (Luo et al. 2022). The total prevalence of bullying in france is at 19%, while 4.9% of the 19% are bully-victims and pure bullies are at 8.9% (Potard et al. 2022). The prevalence of bullying in the Americas is 26.23%. In Africa, the rates are as follows: Ghana, 56.72%; Kenya, 54.35%; Zimbabwe, 59.15%; and Botswana, 52.20% (Man, Liu & Xue 2022). According to statistics from South Africa (Statistics South Africa 2021), 29.2% of secondary learners experience some form of school violence, while the prevalence of bullying is as follows: KwaZulu-Natal 35.9%, Eastern Cape 18.5%, Gauteng 40.1%, and North West 14.6%. Bullying is a repetitive, negative and unwanted act where intimidation or coercion is displayed by one or more learner/s towards other learner/s to display a power imbalance that causes distress and renders them powerless (Park et al. 2022). Bullying is a broad concept as it can occur in different settings, such as in the workplace or at home.

Thus, this study will focus on school bullying, which only occurs within school premises, perpetuated by learners.

School bullying threatens learners’ safety and increases the level of violence in schools (Varela et al. 2020).

Many learners’ lives have been cut short as a direct consequence of bullying perpetration, and this has become a cause for great concern (Chitsamatanga & Rembe 2020). Unsafe school environments, such as a lack of security, can provide fertile ground for bullying, resulting in poor school attendance and injuries from bullying, thereby severely affecting the quality of education and the well-being of learners (Norman 2020).

The following characteristics have been found to expose victims to bullying tendencies: lack of self-esteem, lack of friends, poor social skills, being less popular, sexual orientation, economic status of family, cultural and physical differences, for example, skin colour, gender and disability (McNicholas, Orpinas & Raczynski 2020). Besides, it is highlighted that being different or unique triggers jealousy and envy in other learners, predisposing that learner to bullying acts by peers at school (McNicholas et al. 2020). Furthermore, negative family dynamics such as being neglected and abused, personality traits, for example, aggressiveness and physical appearance of learners can perpetuate the bullying behaviour of adolescents in schools (Wang et al. 2021).

Bullying perpetration and being a victim increase when there is a decreased level of monitoring of learners’ activities and behaviour during breaks and class sessions by teachers in schools (Johansson 2023). For example, monitoring by teachers during breaks and being observant during class sessions were reported to be effective, as bullying occurs in teachers’ absence (Nyawo & Govender 2022).

Bullying does not discriminate, as gender was not found to have a huge role in one becoming a victim of bullying (UNESCO 2023). Nonetheless, both boys and girls can become bullying perpetrators or victims (Chitsamatanga & Rembe, 2020). Consequently, bullying in schools has become a social problem that warrants action from the police, social workers and health personnel to help fight bullying perpetration in schools.

Bullying causes long-term negative psychological effects such as depression, anxiety and physical challenges, for example, sleep deprivation, thus early detection may improve the well-being of learners until adulthood (Lee, Jeong & Roh 2018). A study conducted by Chukwuere et al. (2022) found that the education of learners is negatively affected by depression, anxiety, physical injuries and poor performance in school as a result of frequent bullying acts. In addition to the above paragraph, depression can lead a learner to feel hopeless, unable to cope, have problems at school and be suicidal (Chukwuere et al. 2022). A study by Addy et al. (2021), which focused on mental health difficulties, coping mechanisms and support systems among school-going adolescents, revealed that bullying triggers mental health problems in learners exposed to bullying.

Bullying also results in victims performing poorly academically (Li & Hesketh 2021). Furthermore, UNESCO (2023) reported that bullying has detrimental effects on school attendance, academic performance, mental and physical well-being of learners. Bullying further deprives learners of one of their fundamental human rights, which is the right to education, as it can result in poor academic performance. Thus, it is crucial to tackle and deal with bullying at early stages before it gets out of hand by increasing awareness and monitoring during breaks, also in the classroom by teachers (UNESCO 2023).

Research into coping mechanisms used by bullied survivors in schools around the world has been done but cannot be generalised because of social differences globally and locally (Sittichai & Smith 2018). For example, the study noted that bullying resulted in victims coping by attending poorly at school, reporting to parents or teachers they trusted, avoiding the bully and some fighting back. McNicholas et al. (2020) noted that coping was influenced by family, peer and school support, therefore reducing the negative effects of bullying.

Furthermore, help-seeking, problem-solving and emotion-focused coping, which are regarded as healthy coping strategies, are reported to help reduce the risk of victimisation by helping learners cope with bullying and respond more effectively to such incidents at school (Williford et al. 2022). Teachers, nurses and parents/guardians should have an understanding of why bullying occurs and how learners cope with bullying to identify specific behaviours and remedy the situation timeously (McNicholas et al. 2020). There is limited research into coping mechanisms in the North West province; hence, the researcher deemed it necessary to explore and describe the coping mechanisms used by learners who are bullying survivors using a qualitative research method.

## Research methods and design

A qualitative research approach following exploratory, descriptive and contextual design was used to explore and describe the coping mechanisms used by bullying survivors in this study. This approach allowed participants to give a detailed and in-depth insight into their experience of being bullied and how they coped.

### Research setting

The study was conducted in one secondary school in Dr Ruth Segomotsi Mompati district, Mamusa sub-district in the North West province, South Africa. The specific school was selected as a study site because the researcher had attended assault cases of learners from the same school in the clinic where she works. The interviews were conducted at participants’ homes in the dining room as it was their preferred choice, and the school did not allow for any disturbance of learning activities.

### Population and sampling

The population consisted of 12 learners, seven boys and five girls in grades 9–11 who were between the ages of 14 and 16 years from the selected secondary school. The sample size was determined by data saturation as there was repetition of data. The participants met the inclusion criteria because they were secondary school learners and must have been survivors of school bullying. Purposive sampling was used through the assistance of the school administrator to select the participants who had been bullied at the specific secondary school. The administrator had come across cases of bullying in the school and kept a record as part of administrative duties; hence, purposive sampling was used.

### Data collection

A workshop on bullying was held before the data collection in a hall where one class from grades 9 to 11 learners were invited to attend. The workshop aimed to educate learners about bullying behaviours so they can be able to recognise if they are victims of any form of bullying and also reflect on their roles. Learners received the workshop well, as there was engagement and participation. The workshop lasted for 1 h long. The study is categorised as a medium-risk because there is an increased risk for emotional and psychological discomfort. Therefore, a social worker was part of the workshop to assist with psychological support and was on standby should the need arise or be required by a participant during data collection.

Consent was obtained in written form from parents/guardians and assent from learners identified before data collection by the research assistant. Appointments were made with parents/guardians as well as the participants before data were collected by the researcher at the participants’ homes. As participants are minors, they were approached with a parent or guardian present by the research assistant at their homes. The researcher conducted a pilot study to improve the interview guide and detect ethical concerns, such as the potential to harm participants. The pilot study helps with testing of the interview questions, adjusting the interview schedule accordingly, before embarking on the major research and to assess the researcher’s interview skills (Hui et al. 2022). In this study, a pilot study was conducted with one participant. There were no major changes, and the findings were included in the main study. To facilitate free-flowing conversation and enable participants to provide detailed accounts of their personal experiences, individual in-depth face-to-face interviews were used as a method of data collection. Twelve in-depth individual interviews were conducted, each lasting 30 min – 45 min, until data saturation was reached. Data were collected in Setswana as the setting is in a predominantly Tswana-speaking area, and all participants chose Setswana for data collection. An audio recorder was used to capture the interviews, which were translated verbatim by the researcher. Parents were allowed to sit in during interviews to support their children. During data collection, only one mother was present during the interview out of the twelve conducted to provide support and encouragement. To capture the participants’ contextual meaning of bullying, such as time, place and actions of participants, field notes were taken during the interviews by the researcher.

### Data analysis

Data were analysed using thematic analysis. Thematic analysis steps by Braun and Clarke (2006) used are: (1) the researcher familiarised self with the data by reading, re-reading, writing down ideas and transcribed verbatim, (2) initial codes were generated, (3) generated codes were combined into potential themes, (4) themes were reviewed, (5) naming and refining themes, (6) a report was done on analysed data. All transcripts were meticulously read to write down ideas. After the researcher had finished analysing the data, the co-coder was permitted to do the same and develop themes and sub-themes. To ensure each theme portrayed participants’ views and ideas, themes were reviewed and refined, and both the researcher and the co-coder reached a consensus.

### Trustworthiness

Trustworthiness was ensured by following the five criteria, namely, (1) credibility, which was ensured by prolonged engagement with participants, persistent observation and audit trails. Data were collected over a period of 2 months, each interview lasting 30 min – 45 min. Triangulation refers to the use of different methods to ensure the accuracy and dependability of data collected (Noble & Heale 2019). Triangulation was confirmed by interviewing multiple participants of different age groups, boys and girls at different times. (2) Dependability was ensured by recording interviews and analysing data to ensure comparability, and was achieved by using audit trails and code-recode. (3) Confirmability was ensured by representing data in a way that show participants views and not the researchers viewpoint through audit trail and triangulation. (4) Transferability was achieved through ensuring that the study findings could be applied in another context. (5) Authenticity was achieved through audio recording and verbatim transcription to capture the true feelings and lived experiences of participants. Furthermore, purposive sampling, inclusion and exclusion criteria were used to ensure participants were relevant to answer the research question.

### Ethical considerations

Ethical approval was granded by the Quality in Nursing and Midwifery Scientific Review Committe Scientific Review Committee (NuMIQ SRC), the North-West University Health Research Ethics Committee (NWU HREC), reference number: NWU-00066-23-S1, the Department of Health, North West province (DoH NWP) and the Department of Education, North West province (DoE NWP). Approval was also obtained from the school principal, and consent was obtained from the parents or guardians. Assent and adolescent consent were obtained from the participants before data collection.

Participation was voluntary without any coercion. Participants were informed that they could withdraw at any time without being punished and that they would not be subjected to any harm or stress. Furthermore, participants were treated fairly and equally throughout data collection, ensuring that justice was upheld.

Privacy was ensured by using the alphabet rather than participants’ real names. Privacy at home was ensured as interviews were conducted in the living room with only the participant and the researcher; family members gave space and time for the interview. Respect was upheld by giving information regarding the study so they could make informed decisions on whether to participate or not.

## Results

Themes and sub-themes are described in Table 1.

**TABLE 1:** Summary of themes and sub-themes.

Themes	Sub-themes
1. Positive coping mechanisms	1.1. Resilience 1.2. Extramural activities 1.3. Social support or talking with someone you trust
2. Negative coping mechanisms	2.1. Retaliation by the victim 2.2. Bad habits 2.3. Self-isolation

Two main themes emerged, namely, (1) Positive coping mechanisms, and (2) Negative coping mechanisms, under which six sub-themes emerged as presented in Table 1. Extracts from the in-depth individual face-to-face interviews were used to generate the themes presented.

### Theme 1: Positive coping mechanism

The first theme that emerged is positive coping mechanisms with the following sub-themes: resilience, extramural activities, social support/talking with someone you trust.

#### Sub-theme 1.1: Resilience

The capacity to overcome bullying incidents at school is a common definition of resilience. Resilience was characterised as acceptance of reality and confidence in one’s capacity to manage pressures like bullying.

Therefore, resilience was found to be a skill that two of the participants possessed, and they were able to overcome bullying perpetration.

The following was said to express resilience:

‘I feel ok now, I even forgot about the whole thing. I did not do anything. I would just act like nothing happened and concentrate on the teachers. It did not affect me in any way. I am just fine.’ (Participant number 6, 15 years, Male, Grade 9 learner)

Another participant said:

‘The guy only stopped for two days after we came from the principal’s office. After that, I thought he had changed, but he started it again. I ended up giving him the R2, then the next time I saw him, he started again. I then told myself that I would never give him money again. I told myself that I am not going to be afraid of a person as if it’s something dangerous in front of me, whereas he is a human being just like me.’ (Participant number 7, 16 years, Male, grade 11 learner)

The statements above show that some participants had resilience, which stemmed from believing in their capacity to deal with bullying and acknowledging that they are being bullied. Therefore, resilience protected the victims of bullying from the adverse reactions experienced at school.

#### Sub-theme 1.2: Extramural activities

Extramural activities are referred to as activities performed by learners outside the school curriculum to benefit their personal and academic success, for example, activities such as sports, art and music, among others.

Participants expressed that keeping themselves busy with different activities or sports helped them cope with the bullying perpetration.

The following was said:

‘The time I was bullied, I would hold the fact that I was being bullied to myself and not tell anyone, even at home. I would get home and just go to the gym until the evening. It helped me forget a lot of things happening at school. I would sit with my friends, with whom I play football with full-time, and just talk about football. I would forget about a lot of things.’ (Participant number 2, 14 years, Male, Grade 9 learner)

Another participant said:

‘Eish, I felt otherwise, you know, I don’t know. I would listen to music and watch TV, then I would just sleep. I would also read my books so that I would forget about them.’ (Participant number 3, 14 years, Male, Grade 9 learner)

Football, listening to music and watching TV helped participants deal and cope with the effects of being victimised in school.

#### Sub-theme 1.3: Social support or talking with someone you trust

Social support or talking with someone you trust involves good relationships with friends and parents, which a victim of bullying relies on to be able to cope. A good relationship means good communication, warmth and affection. Parents and friends played a huge role in helping participants deal with the victimisation. One participant said:

‘Yah, they did enough for me [*Parents*]. They would always encourage me to go to school and ignore what the bullies said to me. My parents would say ‘Do not take what they say to heart.’ (Participant number 11, 17 years, Female, Grade 11 learner)

Another participant said:

‘My friends made me forget and told me to relax. To stop thinking of the things I want to do to this guy. When schools reopened, he came to school only once. He saw that when he wanted money from me, I did not give him any attention and didn’t talk to him. I always went to the toilet with my friends.’ (Participant number 7, 16 years, Male, Grade 11 learner)

Another participant echoed:

‘The things I did to forget about him were when my friends made a joke in class, I was able to laugh and make it a joke. I would start forgetting, and they told me to leave him alone. They would always make jokes, and I would forget; even now, I have forgotten about the whole thing. I had forgotten what he did to me because of their jokes. They would make jokes, so I could just forget.’ (Participant number 12, 16 years, Female, Grade 10 learner)

The mentioned quotes demonstrate how social assistance, such as encouragement and support from friends and parents, enabled victims of bullying to deal with the negative repercussions in a positive way.

### Theme 2: Negative coping mechanisms

The second theme that emerged is negative coping mechanisms with the following sub-themes: retaliation by the victim, bad habits and self-isolation.

#### Sub-theme 2.1: Retaliation by the victim

Retaliation is the act of harming the bully because they have harmed the victim first. Participants in the study reported retaliating by using violence as a way of coping with the bullying. The following was expressed regarding this notion:

‘They were not aware, but they ended up seeing for themselves the day I was fighting with the guy. So they asked me what was happening with me and the guy. I then told them that each and every day I went to the toilet, I must give him R2. They started being aware and kept an eye on him. They also saw when the teacher who took us to the principal approached me and started realising that what I had told them was true.’ (Participant number 7, 16 years, Male, Grade 11 learner)

Another participant said:

‘They wanted to take my book while I was writing. They wanted to make fun of me, making me a laughing stock in class. So I ended up losing my temper and I hit them.’ (Participant number 11, 17 years, Female, Grade 11 learner)

Bullying resulted in victims retaliating against their bullies out of frustration and to stop the bullying perpetration. The participants were greatly affected by the experience of bullying perpetration, resulting in victims resorting to physical violence and fighting. The act of fighting was an attempt to push away their bullies or a way of protecting themselves from the bullies.

#### Sub-theme 2.2: Bad habits

Actions or behaviours that victims of bullying do that are harmful to them but provide temporary relief are said to be bad habits. Harmful behaviour was displayed by some of the participants, which was used as a way to cope with the victimisation. The following was said to support this notion:

‘I then started befriending boys on the next street as they smoked a hookah pipe, so I started smoking it. I saw that it would take me out of a lot of bad things. As time went on, we learnt that the lungs of one of the boys were filled with water, so I left that group and started staying in the house because these boys made me smoke this hookah pipe as they were always on my mind.’ (Participant number 5, 16 years, Male, Grade 9 learner)

Another participant said:

‘I wanted to take the knife he took out for me to stab him with it and see what would happen to him after I stabbed him. To see how he would feel. Would he stop what he was doing? But the guy ended up dropping out of school. I don’t know if I took him out of school or what.’ (Participant number 7, 16 years, Male, Grade 11 learner)

The participants resorted to bad habits as a way to try to cope with the situation at school.

#### Sub-theme 2.3: Self-isolation

Self-isolation is an act of staying away/avoiding learners/teachers and being alone most of the time. Participants isolated themselves as a way of coping, and this was expressed as follows:

‘I always play with my siblings; I would read stories and watch movies. As time went by, I forgot about the bullying. At school, I would just sit still in class and even end up not going for a break.’ (Participant number 11, 17 years, Female, Grade 11 learner)

Another participant said:

‘When I see them, sometimes I just sit at home, or when I see them from a distance, I run away/I do something/I change paths/I lock myself in the house, and wait for them to pass. That’s what I always do.’ (Participant number 8, 14 years, Female, Grade 9 learner)

Another participant reiterated:

‘I would feel sad, scared, and I would hide when I saw them so that they would not see me. Because if they saw me, eish! There would be a problem.’ (Participant number 3, 14 years, Male, Grade 9 learner)

The participants ended up in self-isolation because they were afraid of being targeted by the bullying perpetrators.

## Discussion

The increasing phenomenon of bullying in schools results in victims finding ways to cope and deal with their bullies. The positive coping mechanisms displayed by victims are resilience, extramural activities, and talking to a trusted person. The finding is affirmed by Riley et al. (2020) that different coping mechanisms used by victims of bullying have different results, which can be positive or negative. Furthermore, the well-being of victims of bullying is affected regardless of the coping mechanisms used. It is also noted that the use of positive coping mechanisms tends to give victims control over the bully and increases confidence in victims. In concurrence, negative coping mechanisms affect victims’ mental health negatively (Kolesnyk et al. 2020).

Farchi and Peled-Avram (2025) affirm that resilience is characterised by personal traits that include personal qualities such as belief in one’s ability, having hope, strength and confidence. Furthermore, it is stated that resilience is shaped by early-life experiences such as safe family environments and unconditional support from family. However, for this study, it was not determined how they came to be resilient, and further research is needed for this concept. Resilience is said to be a supportive factor against mental health problems in victims of bullying (Harrison, Loxton & Somhlaba 2021). As shown by two of the participants reporting that they were not affected by the victimisation and that they just coped well with the bullying they experienced.

Findings further revealed that playing soccer, listening to music and watching TV were used to cope with bullying. A study conducted by McNicholas et al. (2020) established that participating in activities such as sports could boost the confidence of victims of bullying. Shumba, Nyambuya and Khumalo (2023) affirm that music increases feelings of happiness and can help one deal with stressful situations. Furthermore, music heightens feelings of happiness and boosts the self-esteem of victims. Positive coping mechanisms can benefit victims by functioning as a protective factor, reducing bullying and increasing the well-being of affected individuals (Riley et al. 2020). Some participants indicated that they were able to cope with being bullied through the support they received from friends and family. McNicholas et al. (2020) assert that family and peers can help victims cope effectively through the support they give. Participants reported that friends played a huge role in assisting them to cope with the bullying by uttering words of encouragement and making jokes. One participant reported that parents also played a huge role in helping him cope by encouraging and supporting him. According to Zhang et al. (2016), social support shields bullied individuals by providing a feeling of belonging and warmth, therefore improving their mental health.

Negative coping mechanisms result in the inability of victims to effectively deal with bullying perpetration by acting out and ignoring the bully (Riley et al. 2020). In this article, negative coping mechanisms resulted in a poor change of behaviour and worsened the effects of bullying. Participants revealed that they retaliated out of frustration and as a way of protecting themselves or attempting to stop the bully. Most participants reported losing their temper because of the frustration of being constantly bullied and therefore wanting to hurt the bully/ies. Potard et al. (2022) assert that victims of bullying display aggressive behaviour and want to retaliate in order to compensate for feelings of shame and powerlessness they felt when they were bullied. One participant reported fighting back because bullies wanted to turn her into a laughingstock in class. Bitsika and Sharpley (2021) support the findings by asserting that, to protect themselves from imminent danger, victims of bullying would confront the bully or talk back. Learners who are exposed to bullying at school are subjected to some form of bad behaviours (Foody & Samara 2018). According to Kolesnyk et al. (2020), bad habits are behaviours such as smoking, taking part in harmful activities such as fighting and aggression. Victims reported that this whole experience greatly affected their emotions and would get angry and want to stab the bully, while another participant resorted to smoking in an attempt to suppress the negative emotions. These findings are corroborated by Foody and Samara (2018:3), who assert that learners who are exposed to bullying develop emotional and psychological problems. Li and Hesketh (2021) concur with the findings by noting that victims of bullying suffer emotional distress because of continued bullying and the embarrassment they feel from the ordeal.

Participants reported isolating themselves as a way of coping with bullying and to avoid the bully because they feared being hurt or humiliated in public. The findings are supported by Harrison et al. (2021), who assert that victims of bullying self-isolate in order to avoid the bully. Likewise, victims of bullying are said to self-isolate as a way of limiting exposure to bullies and reframing thoughts to try and forget about the bullying experience (Parikh et al. 2019). This form of coping can put victims of bullying in great danger of not reacting well to the demands of that situation, which in turn can exacerbate the problem. Chitsamatanga and Rembe (2020) concur that school bullying is said to cause insecurities in victims of bullying, hence they isolate themselves from others as they believe they are easy targets. One participant said that she would not go out during breaks at school and sit still in class. Varela et al. (2020) highlighted that the quality of life for victims of bullying was very low/poor as a result of experiencing bullying in schools. This is mainly because of fear of being hurt or humiliated by their bullies in public spaces, which may further result in negative coping mechanisms, as indicated by participants who felt sad and scared.

### Limitations

The limitations of this research were that only learners from the specific school, predominantly speaking Setswana, took part in the study. This was a qualitative study; therefore, generalisation of findings is limited. Further limitations are that only learners in grades 9–11, between the ages of 14 and 16 years, were included in the study.

## Conclusion

Findings from the study show that bullying affects victims emotionally, psychologically and physically.

Participants in this study coped using positive and negative mechanisms, which affected victims in their respective ways. The findings further suggest that victims who used positive coping mechanisms had improved their mental health and well-being as opposed to those who used negative coping mechanisms. How victims coped with the victimisation determined their psychological and emotional well-being. Therefore, extensive awareness and education on bullying needs to be emphasised in schools for teachers, parents/guardians, nurses, police and the school governing bodies to effectively deal with school bullying and identify the victims early. Furthermore, education on bullying needs to be incorporated into nursing education for nurses to be knowledgeable on risk factors, coping mechanisms used by victims and management.
